# Supplementary figures and images for: The HTLV-1 viral oncoproteins Tax and HBZ reprogram the cellular mRNA splicing landscape
Source: PLoS Pathog. 2021 Sep 20;17(9):e1009919. doi: 10.1371/journal.ppat.1009919 (PMC8483338; doi:10.1371/journal.ppat.1009919)

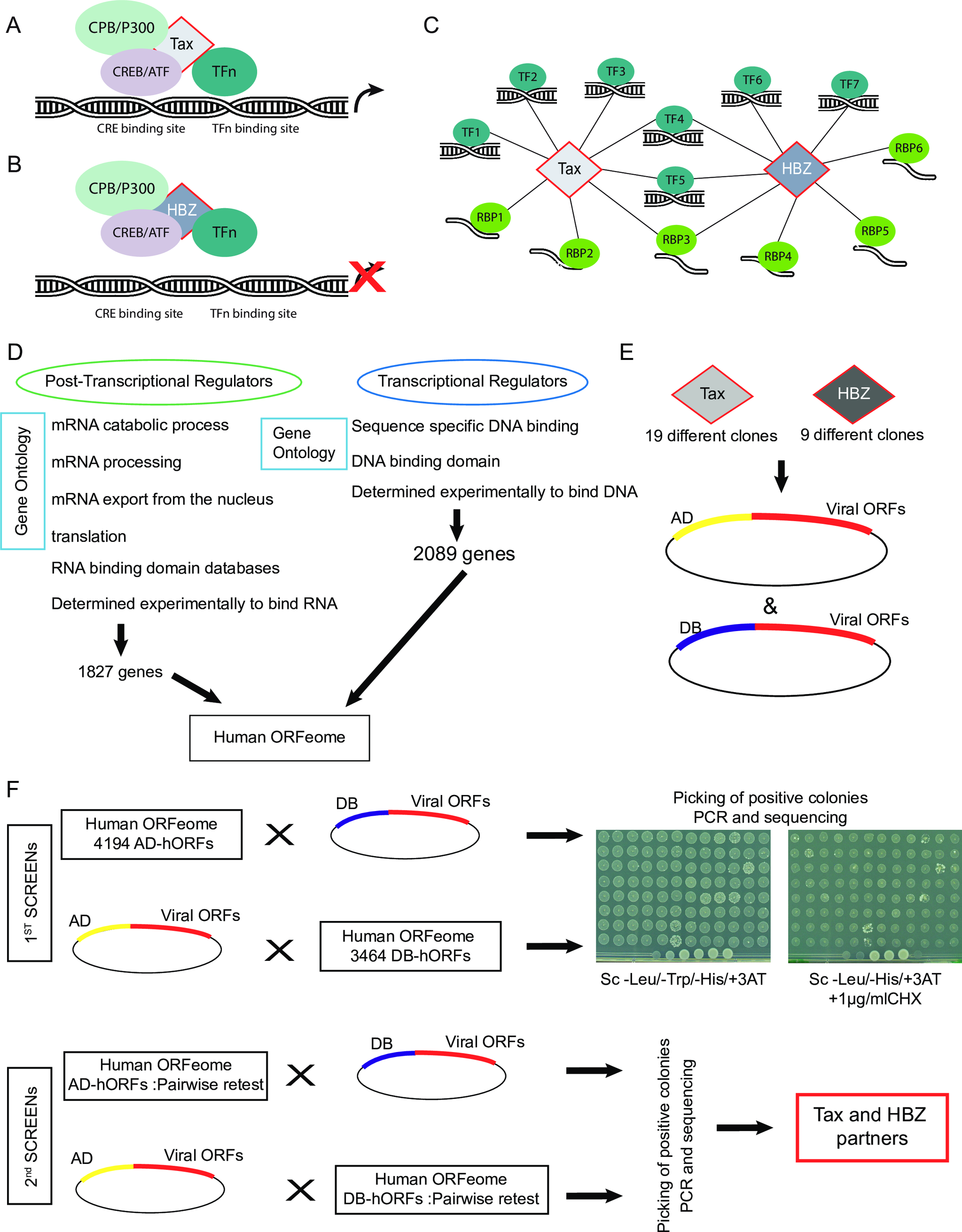

Supplement: S1 Fig — (A) Schematic representation of positive interaction between Tax, CREB/ATF and CBP/P300 on the viral promoter. (B) As in (A) but negative interaction driven by HBZ. (C) Illustrative network diagram showing interactions between Tax and HBZ with cellular transcription factors (TF) or RNA-binding proteins (RBP). (D). Pipeline to generate a comprehensive list of human RBPs and TFs. (E) Cloning strategy for the Tax/HBZ mini-library. (F) Y2H strategy to identify high-quality protein-protein interactions (PPIs). (TIF) [file ppat.1009919.s001.tif]

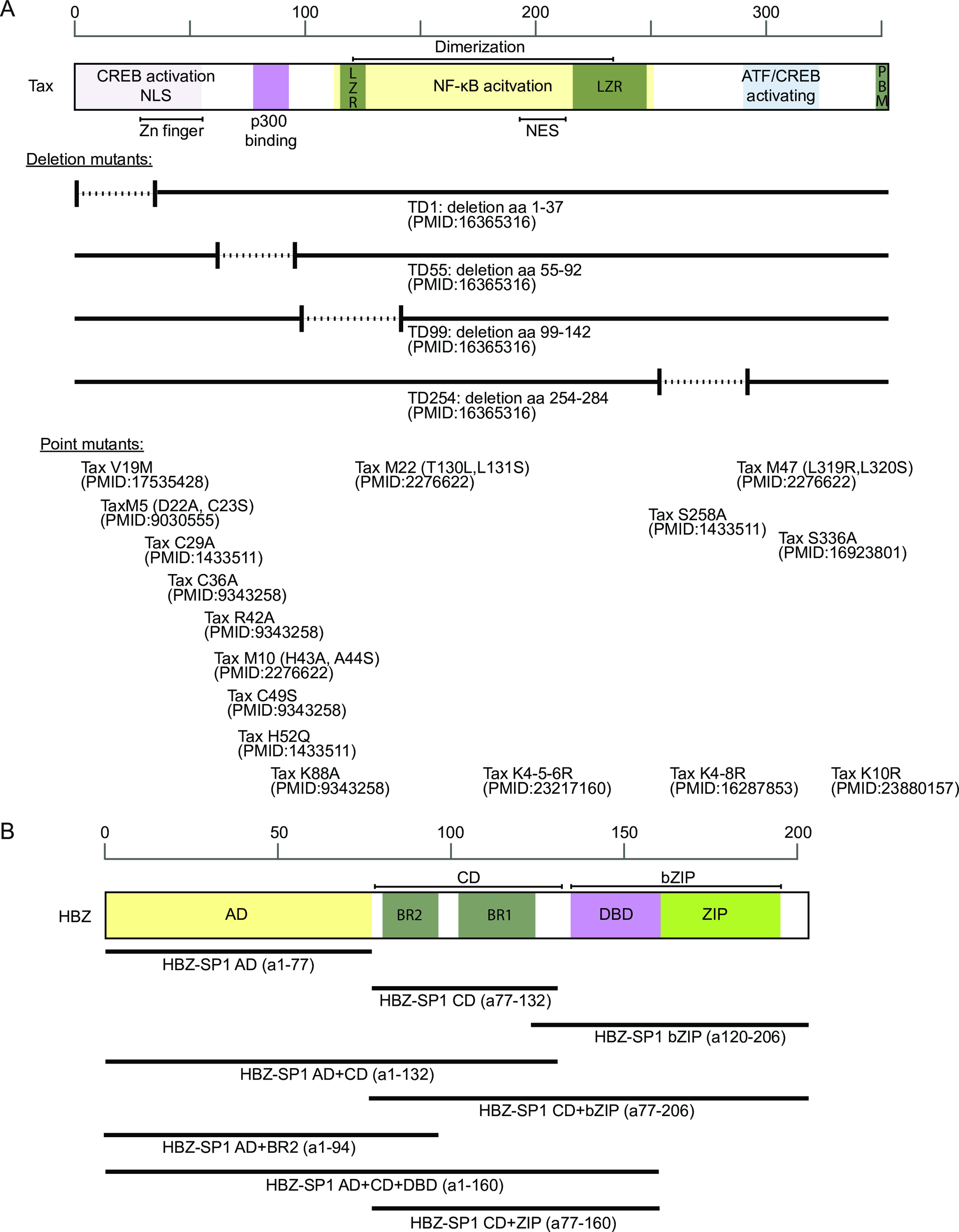

Supplement: S2 Fig — Representation of the functional and structural domains of (A) Tax and (B) HBZ. (A) Tax deletion and point mutants used for Y2H screening are described below Tax diagram, with references as PMIDs. (B) HBZ deletion mutants used for Y2H screening are depicted below the diagram. NLS = nuclear localization signal, NES = nuclear export signal, LZR = leucine zipper-like motif regions, PBM = PDZ domain binding motif. (TIF) [file ppat.1009919.s002.tif]

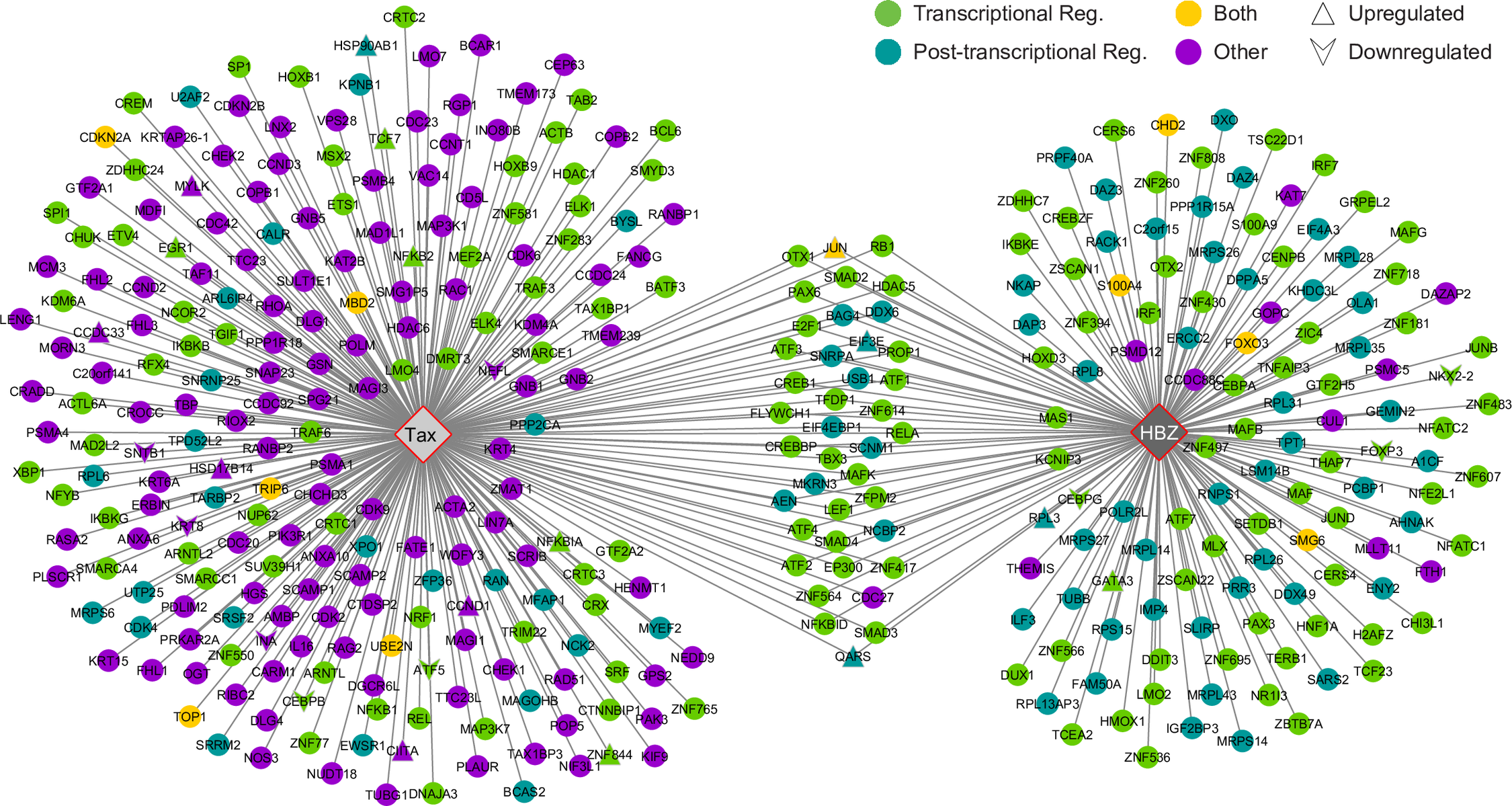

Supplement: S3 Fig — Host proteins are color-coded according to their function in Transcriptional Regulation (TR), Post-Transcriptional Regulation (PTR) or other (purple). Upward triangles and downward arrows show genes with an up-regulation or down-regulation following Tax or HBZ expression, respectively. (TIF) [file ppat.1009919.s003.tif]

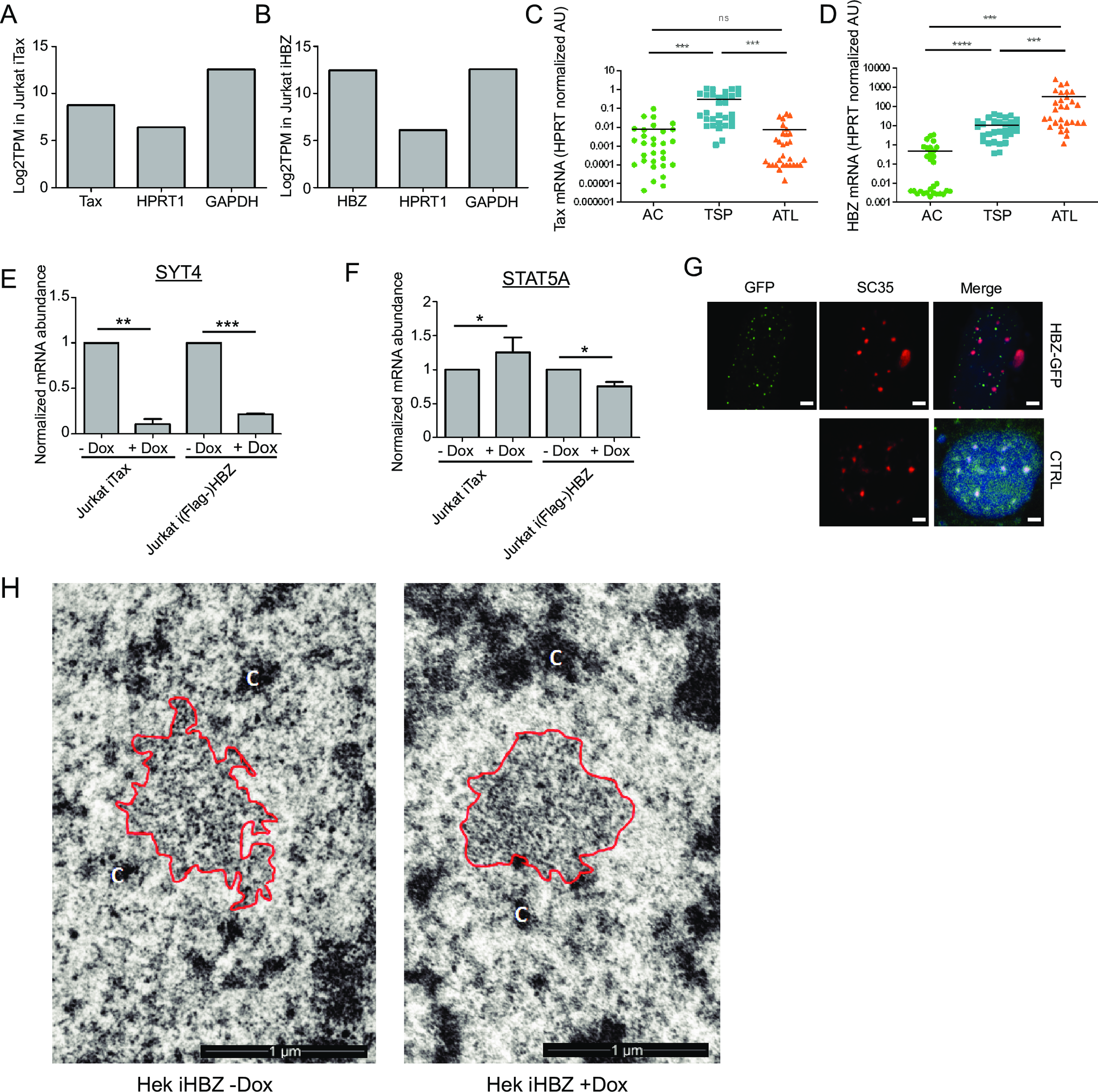

Supplement: S4 Fig — (A-B) Log2TPM of Tax, HPRT1, GAPDH and HBZ mRNA expression in Jurkat-iTax (A) or Jurkat-i-HBZ (B). (C-D) Normalized Tax and HBZ mRNA expression in ATL, TSP and asymptomatic carrier (AC) samples. qPCR data are normalized to the expression of HPRT1 mRNA. (E-F) qRT-PCR analysis showing variation of normalized mRNA abundance of (E) SYT4A and (F) STAT5A upon expression of Tax and HBZ. * = p-value <0.05, ** = p-value <0.01, *** = p-value <0.001. (G) Immunofluorescence microscopy indicates that SC35, a marker of nuclear speckles, localizes into more round shapes upon expression of HBZ in HeLa cells. Scale bars = 2 μm. (H) Representative nuclear portion of HEK293T expressing HBZ(left) or not (right), visualized by TEM. Clusters of interchromatin granules (also known as nuclear speckles) are surrounded by red dotted lines and display a more compact phenotype upon HBZ expression. C = condensed chromatin. Scale bars = 1 μm. (TIF) [file ppat.1009919.s004.tif]

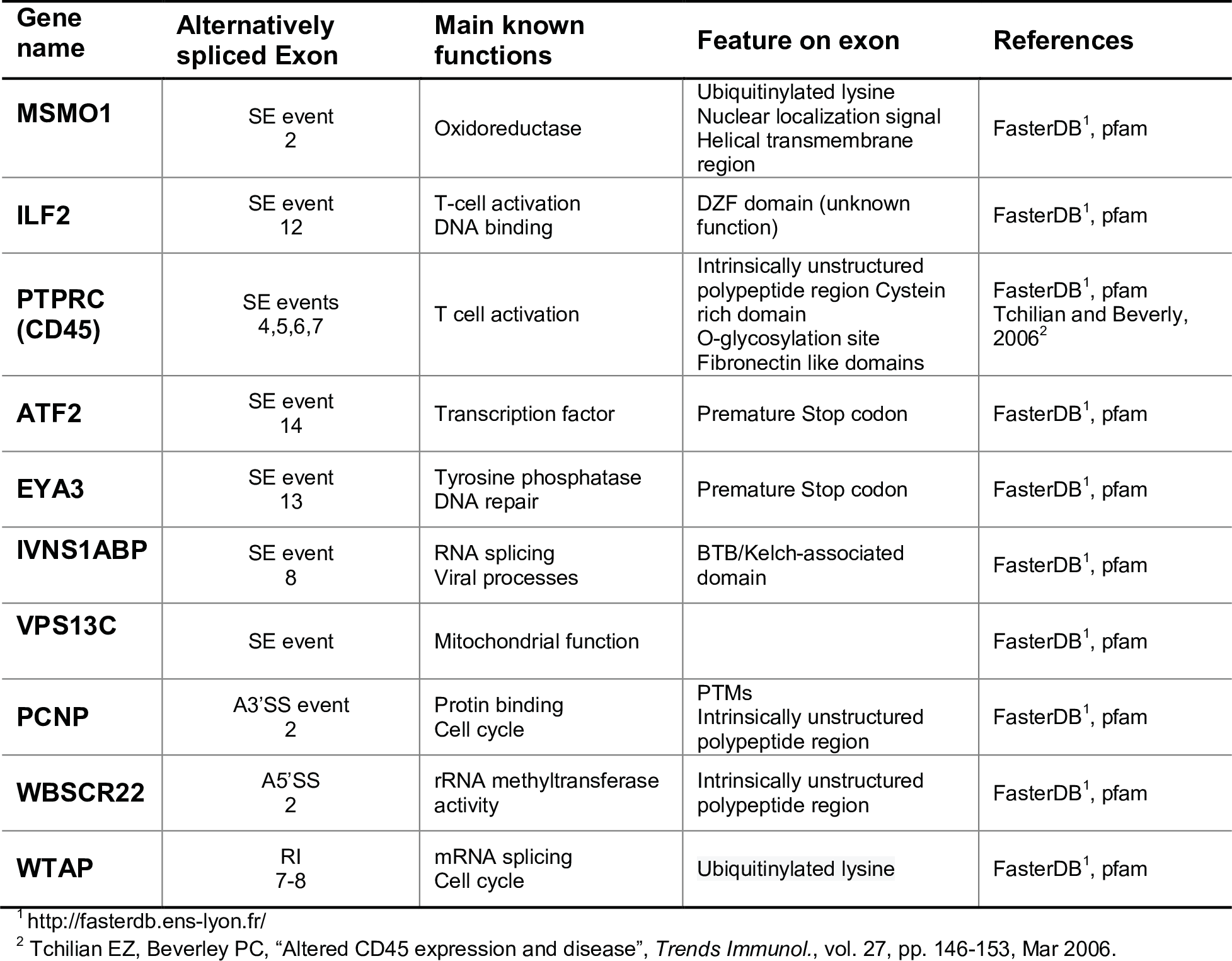

Supplement: S5 Fig — (TIF) [file ppat.1009919.s005.tif]

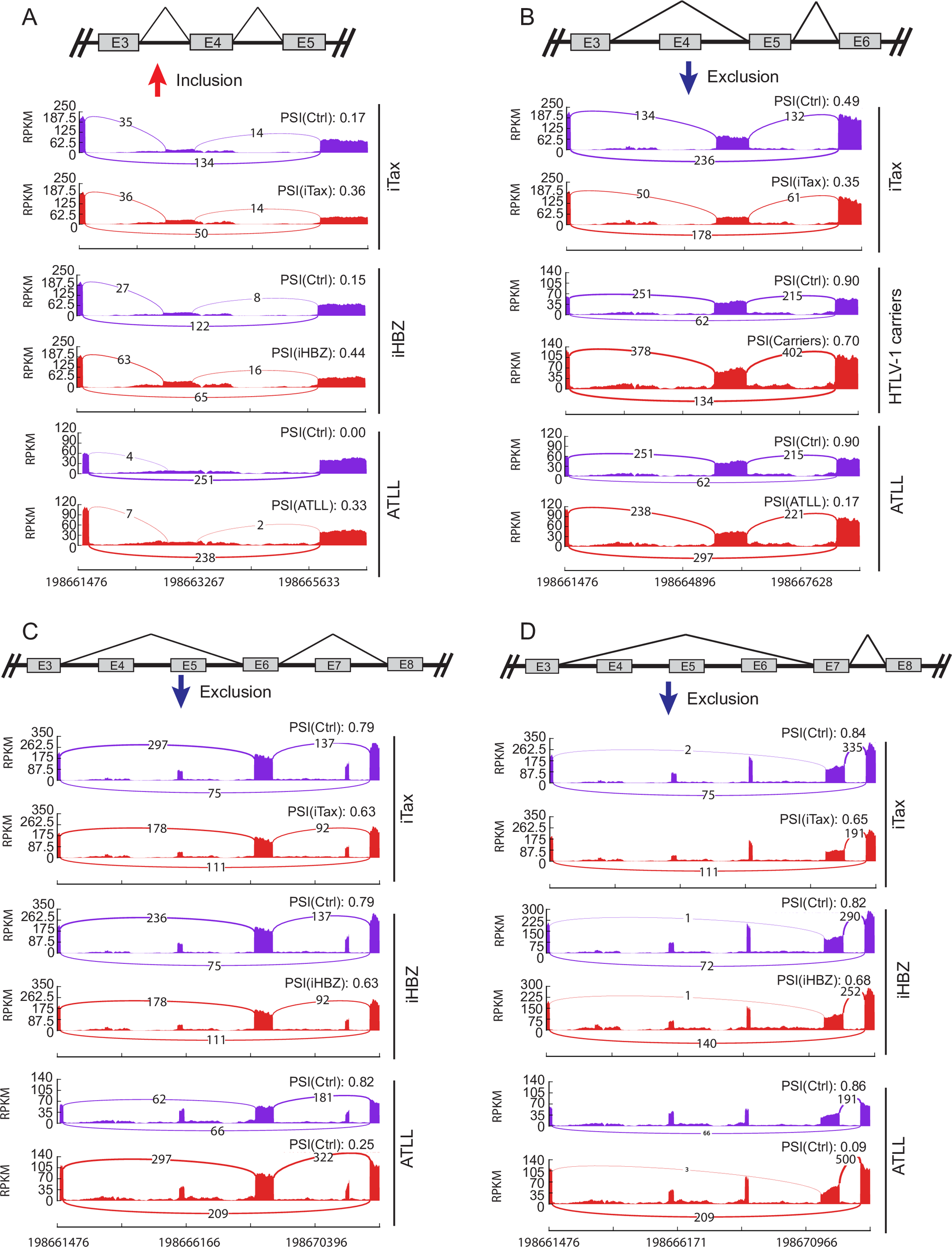

Supplement: S6 Fig — Splicing events are depicted above each sashimi plot, coordinates of regulated and flanking exons are indicated at the bottom. (A) Increased inclusion of exon 4. Decreased inclusion of exon 5 (B), 6 (C) and 7 (D). For ATLL, sashimi plot is a representative case. (TIF) [file ppat.1009919.s006.tif]

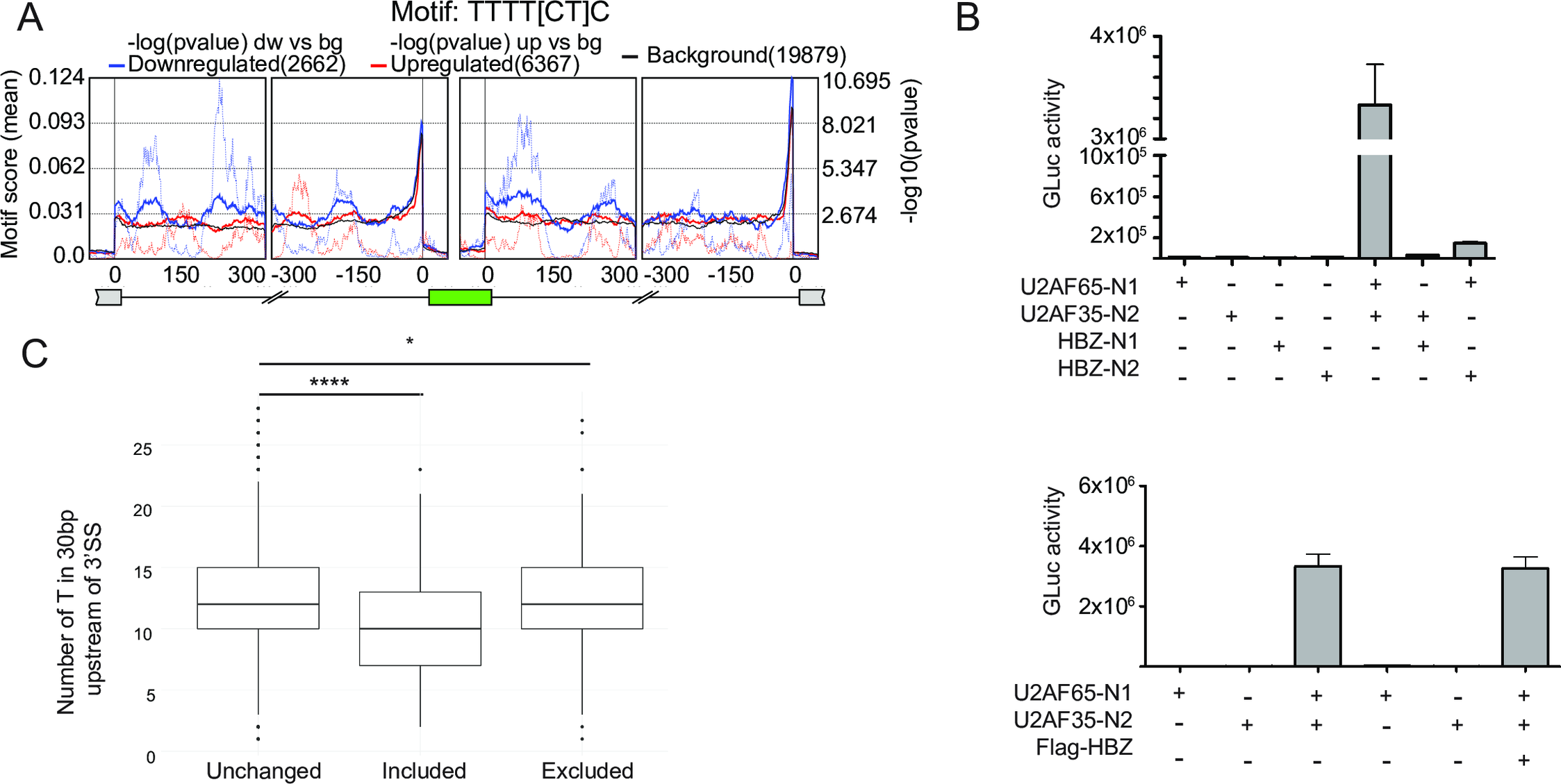

Supplement: S7 Fig — (A) Solid lines indicate the mean U2AF2 binding motif score calculated in a 50 bp sliding window. Dotted lines indicate -log10 p-values obtained by statistical comparison of motif scores between modified exons (exclusion = down-regulated and inclusion = up-regulated) against non-modified background exons. Green box represents regulated exons flanked by neighboring introns and upstream and downstream exons in black lines and grey boxes. (B) GPCA to test the absence of interaction between HBZ and U2AF complex subunits (U2AF65 and U2AF35). Y-axis shows luciferase activity for a representative experiment of 3 repetitions. (C) Number of Ts in the 30bp upstream of 3’SS of alternatively spliced exons (SE events) in Jurkat-iHBZ cells. Medians are represented by a line. Included P <2.2e-16, Excluded P = 0.03398, by Welch Two Sample t-test in R. (TIF) [file ppat.1009919.s007.tif]
