# Supplementary material for: The HTLV-1 viral oncoproteins Tax and HBZ reprogram the cellular mRNA splicing landscape
Source: PLoS Pathog. 2021 Sep 20;17(9):e1009919. doi: 10.1371/journal.ppat.1009919 (PMC8483338; doi:10.1371/journal.ppat.1009919)
Supplement: Attachment — Submitted filename: Responses to reviewers_Vandermeulen et al.docx [file ppat.1009919.s012.docx]

# **Responses to reviewers (in blue below each comment)**

## I. Editorial comments:

Thank you very much for submitting your manuscript "The viral oncoproteins Tax and HBZ reprogram the cellular mRNA splicing landscape" for consideration at PLOS Pathogens. As with all papers reviewed by the journal, your manuscript was reviewed by members of the editorial board and by several independent reviewers. In light of the reviews (below this email), we would like to invite the resubmission of a significantly-revised version that takes into account the reviewers' comments.

All three reviewers found your study of interest, novelty and potential importance. However, all three also raised significant issues that need to be addressed. The most important points concern 1. the relationship between Jurkat cells and primary T cells, 2. the difference (if any) in the level of expression of Tax and HBZ between the inducible Jurkat cell lines and primary cells (whether non-malignant or ATL cells), and 3. the need to balance the numbers of control samples with the experimental samples. In addition, the reviewers asked for more details on the methods used, in particular the bioinformatic analysis.

We cannot make any decision about publication until we have seen the revised manuscript and your response to the reviewers' comments. Your revised manuscript is also likely to be sent to reviewers for further evaluation.

We thank you for your interest in our paper and appreciate the thoughtful review process. Below we respond to each of the most important points raised by all the reviewers, and as a result the revised manuscript has been significantly improved.

1. The relationship between Jurkat cells and primary T cells,

Jurkat is a T-acute lymphoblastic leukemia cell line established from the peripheral blood of a 14-year-old boy in 1976 by [Schneider](https://pubmed.ncbi.nlm.nih.gov/?sort=date&term=Schneider+U&cauthor_id=68013) et al (Int J Cancer. 1977 May 15;19(5):621-6). Jurkat cells are CD2, CD3, CD4, CD5, CD6, CD7 and TCRalpha/beta positive; and CD8, CD13, CD19 and TCR gamma/delta negative. Primary human T cells can be isolated from peripheral blood mononuclear cells (PBMCs) of healthy donors. Based on age, ethnicity, prior exposure to pathogens, primary T cells will contain variable abundance of different subsets of T cells (CD4+, CD8+, naive, memory,...), which is also the case in both ATL patients and asymptomatic carriers in this study (see below).

Moreover, primary T cells need exogenous activation (using anti-CD3, anti-CD28, IL-2) to proliferate, whereas Jurkat cells produce IL-2 and are more easily and rapidly expanded in culture. These practical reasons explain our choice of the Jurkat cell line to study the perturbations of gene expression following Tax and HBZ expression.

The Jurkat model system has contributed significantly to our understanding of T-cell activation and T-cell receptor engagement, calcium signaling in T-cells,... (Abraham and Weiss, [*Nature Reviews Immunology*](https://www.nature.com/nri) volume 4, pages 301–308 (2004); Meuer, S. C. et al., *Nature* 303, 808–810 (1983)), and, of relevance for this work, the life-cycle of human retroviruses, HIV-1 (1568 publications in Pubmed including 59 since 2020), and HTLV-1 (448 publications including 13 since 2020), is still a valid model for mechanistic understanding of viral-host interactions in T cells .

As stated in our discussion section (page 14, lines 9-12), although the Jurkat experimental system does not take into account the level of expression and dynamic interplay of viral proteins *in vivo*, it does allow for a systematic and unbiased comparative analysis to identify Tax- and HBZ-dependent events contributing to dysregulation of cellular function.

2. The difference (if any) in the level of expression of Tax and HBZ between the inducible Jurkat cell lines and primary cells (whether non-malignant or ATL cells),

We thank you for this important question, particularly when comparing data obtained following Tax and HBZ expression in inducible systems versus primary HTLV-1+ cells.

To estimate the relative expression of Tax and HBZ in our Jurkat cells, we used our RNA-seq data and computed the average Log2TPM of Tax, HBZ and a reference host gene (hypoxanthine-guanine phosphoribosyltransferase-1 (HPRT1)). We obtained the following results:

**Jurkat-i-Tax**

Log2TPM Tax :8.77

Log2TPM HPRT1 : 6.4

Relative Tax expression: 1.37

**Jurkat-i-HBZ**

Log2TPM HBZ : 12.47

Log2TPM HPRT1: 6.11

Relative HBZ expression: 2.04

We compared our RNA-seq quantifications to qRT-PCR data obtained from ATLLs of an Afro-Caribbean cohort (Dr. Peloponese, CNRS-Montpellier France), where the average relative *Tax* and *HBZ* mRNA expression were 0.01 and >100 respectively (see figure below, C and D). Doing so, we found that the expression of both viral gene mRNAs were 137-fold higher for *Tax* and 50-fold lower for *HBZ* in inducible Jurkat cells compared to primary ATL cells. As shown in Figure S4 below, these magnitudes were also observed within each category of individual patient samples (HTLV-1 carriers, ATL and TSP (Tropical Spastic Paraparesis) patients. The expression of *tax* and *hbz* transcripts in ATLs was also analyzed using RNA-seq data of the Afro-Carribean cohort used in the paper (reported in Rosewick et al 2017 and Marçais et al 2021). Tax is rarely expressed and if it is, the levels are extremely low. Regarding *hbz*, it is consistently expressed in all primary ATL samples, but with strong inter-patient variability.

Figure S4A-D legend: (A-B) Log2TPM of *Tax*, *HPRT, GAPDH* and *HBZ* mRNA expression in Jurkat-iTax (A) or Jurkat-i-HBZ . (C-D) Normalized Tax and HBZ mRNA expression in ATL, TSP and asymptomatic carrier (AC) samples. qPCR data are normalized to the expression of HPRT1 mRNA.

3. The need to balance the number of control samples with the number of HTLV-1-positive samples.

We thank the reviewer for raising this important issue. We agree that, similar to the majority of published studies using HTLV-1+ patient cohorts, our work employed a relatively low number of healthy controls compared to diseased samples. CD4+ T-cells from healthy volunteers are often used (for example 3 samples in Kataoka et al. Nat. Genet. *47*, 1304–1315; and 4 samples in Rosewick et al. Nat. Commun.*8*, 15264.) as controls and do not reflect the heterogeneity seen in ATL cases. Longitudinal analyses, from HTLV-1 seroconversion to ATL induction and progression, would be ideal to identify significant molecular changes within individual patient samples over time. In the absence of such samples, and inspired by your question, we analyzed and compared the above referenced cohorts from different ethnicity groups of patients. In the revised version, we added an Afro-Caribbean cohort comprising ATLs, asymptomatic infected individuals, and also healthy volunteers. Our rationale is that common key molecular features driven by Tax or HBZ expression, might be observed in malignant ATL cells regardless of the patient' ethnicity or geographical origin. This comparative analysis in reasonably large independent cohorts may thus provide candidate molecular biomarkers for ATL, and possibly guide future ATL therapeutic strategies.

We thus analyzed, separately, alternative splicing events (ASEs) comparing ATLs, HTLV-1 asymptomatic carriers and healthy individuals from the Japanese (Figure 5), and the Afro-Caribbean (Figure 6) cohorts. From this analysis, we were able to draw three important conclusions: (i) in both cohorts, spliced exons (SE) and mutually exclusive exons (MXE) accounted for a large majority of ASEs (compare Fig 5A and Fig 6A), (ii) a number of ASEs from both cohorts are also identified in Jurkat cells expressing either *Tax* or *HBZ* genes (compare Fig 5C and Fig 6C) and (iii) we have identified common ASEs in both patient cohorts that also overlap with *Tax-* or *HBZ-*driven splicing events (Fig 7A). Genes impacted by ASEs in both cohorts are compiled in Table S3, sheet 18.

We have added, in the discussion section, a paragraph highlighting the above study limitations (Page 14, lines 21-31).

**Part I - Summary**

**Reviewer#1:** The authors have screened interacting host factors binding with HTLV-1 Tax and HBZ by using Y2H system. They identified distinct but also common host factors between Tax and HBZ. They further performed comparative analysis of transcriptomic changes associated with Tax and HBZ using Jurkat with HBZ- and Tax-inducible cells. They found Tax and HBZ has an impact on cellular gene alternative splicing events. The aberrant alternative splicing events were found in ATLL patients. Tax and HBZ interact with RNA processing factors, such as U2AF2, and thereby inducing alternative splicing events.
The findings shown are potentially interest contain some novelty.

We appreciate and agree with these comments.

However, some figures are difficult to understand and also there is limited description about the bioinformatic method used. Some data presented seems to be preliminary and not so convincing. It would be better to add more explanation and evidence to support authors’ proposed title “The viral oncoproteins Tax and HBZ reprogram the cellular mRNA splicing landscape”.

We appreciate this reviewer's summary: We have revised the manuscript by adding new figures, including Figure 6 that complements Figure 5 to validate our findings that Tax and HBZ are associated with alternative splicing events and that such events are also observed in two independent patient cohorts from Japanese and Afro-Caribbean origin. Mechanistically, we showed Tax and HBZ interact with a number of RNA binding proteins including the U2AF complex, a key regulator of pre-mRNA splicing (Figures 1 and 8). Importantly we showed that Tax could stabilize the interaction between U2AF1 and U2AF2 (Figure 8F-G), with similar ASEs found in Tax expressing cells as well as in cells knocked-down for U2AF2 (Figure 8 I-O). More explanation is provided in the discussion as suggested by this reviewer.

In addition, our RNA-seq data analysis section is now more detailed (Page 23, lines 22-33; Page 24, lines 1-8) as well as the legend of Figure 3A.

**Reviewer #2**: In the present study, Vandermeulen investigated the effects of Tax and HBZ on the transcriptome and interactome in Jurkat cells. Results uncovered many interactions with RNA-binding proteins, including U2AF2, a key cellular regulator of pre-mRNA splicing. Consistent with this finding, Tax and HBZ perturbed the splicing landscape of Jurkat cells. Interestingly, Tax favored exon inclusion while HBZ induced exon exclusion. A subset of Tax- and HBZ-dependent splicing changes were also found in cells from ATLL patients. The study is potentially interesting and investigates the effects of 2 key HTLV-1 proteins (Tax and HBZ) on a poorly explored field – i.e. the regulation of the host RNA splicing by viral factors.

We thank the review for these comments.

**Reviewer #3:** Vandermeulen et al. in their manuscript entitled “The viral oncoproteins Tax and HBZ reprogram the cellular mRNA splicing landscape” analyzed the transcriptome and interactome of the HBZ and Tax viral proteins. The authors found that each protein has distinct targets, but also share common transcription factors and RNA-binding proteins. By analyzing the cellular splicing landscape using rMATS software, HBZ and Tax were found to alter the splicing landscape in T-cells. Interestingly, they exert opposing effects with Tax inducing exon inclusion and HBZ inducing exon exclusion. Several of the splicing changes identified by the authors were also altered in ATL patients. The manuscript was well-written and is of high importance to both the HTLV-1 field and other oncogenic or pathogenic viral fields. However, several comments/suggestions are provided below for the authors’ consideration in order to satisfy the PLOS Pathogens criteria for publication.

We thank the reviewer for highlighting the importance of this study in the field. We have addressed reviewers’ comments below.

### Part II. Major issues:

Reviewer #1:

1.Alternative splicing analysis. Fig. 3A
It is not well explained in current manuscript about how they define alternative splicing events. In the method, “ASEs were filtered to consider only events with a ΔPSI >1 or <-1 and with a FDR <0.05”. Just a few percent variation of PSI can be caused by experimental replication. The authors need to explain more about data representativeness. How many replicates they performed for RNA-seq analysis, for example (Jurkat and patient samples). It is difficult to understand Fig. 3E. How did they make this graph? In general, RT-PCR may not be good to distinguish small change of cDNA amount. ddPCR would be useful for the purpose. In Fig. 7 L-O, there were only less than 1 percent difference in PSI by RT-PCR. It is hard to say there is differences. I may misunderstand the graph, so please explain more in details.

We apologize for the lack of clarity regarding the “Percent Spliced In” calculation and the error while defining its threshold in the text. rMATS first calculates the PSI for each condition and replicates, and then reports the ΔPSI between the two conditions. ΔPSI is a value ranging between -1 and +1. We have corrected this in the text, in the legend of the Figure 3A and in the methods. Fig. 3E from the previous version is now Fig. 4A (shown below), and it is presented as bar graphs for more clarity as suggested by this reviewer. We show, side-by-side, ΔPSI calculated from RNA-seq data using rMATS and ΔPSI calculated from RT-PCR validation experiments. 22 out of 23 tested spliced events exhibited a similar tendency of splicing (inclusion or exclusion).

**Previous Fig. 3E**

**Revised Fig. 4A**

2. Figure 4
RNA transcription and splicing are basically distinct biological processes, even though there is some relationship between them. Thus, we can expect the observation that only a small number of genes (23 and 29 for Tax or HBZ, respectively) presenting ASEs were differentially expressed (Fig. 3 C and D). It is not clear for me that the results is valuable to show as figure.

Whereas transcription and post-transcription require distinct multi-component molecular machines, there is increasing evidence that, rather than sequential events, RNA transcription and processing are coupled biological processes, interconnected in regulatory circuits, to ensure quality control of the entire gene expression pathway (for review see Rambout et al. Chem Rev. 2018 Apr 25;118(8):4339-4364; Maniatis and Reed Nature. 2002 Apr 4;416(6880):499-506; Brody and Tav-Tal, [Transcription.](https://www.ncbi.nlm.nih.gov/pmc/articles/PMC3265778/) 2011 Sep-Oct; 2(5): 216–220.). As an example, we have previously demonstrated that mRNA synthesis driven by the ERG transcription factor is coupled to degradation through the recruitment of the CCR4-NOT deadenylation complex (Rambout et al.Nat Struct Mol Biol 2016 Jul;23(7):663-72). Regarding splicing, it is now accepted that the spliceosomal machinery assembles on nascent transcripts while still tethered to the actively transcribing RNA polymerase, explaining why the majority of splicing events occur ***co-transcriptionally***. (For detailed examples: Osheem et al. Cell. 1985 Nov;43(1):143-51.; Rambout et al. Chem Rev. 2018 Apr 25;118(8):4339-4364; Brody and Tav-Tal, [Transcription.](https://www.ncbi.nlm.nih.gov/pmc/articles/PMC3265778/) 2011 Sep-Oct; 2(5): 216–220).

Our observation that only a small number of genes (23 and 29 for Tax or HBZ, respectively) presenting ASEs were differentially expressed is unexpected and suggest that Tax and HBZ might favor ***post-transcriptional*** RNA processing, as a mechanism to destabilize global gene expression quality control. Following this reviewer comment, we present this global effect in Fig. 4D, and highlight genes that are ***co-transcriptionally*** spliced (Fig.4E).

**Previous Fig. B and C**

**Revised Fig. 4D-E**

3. Figure 5
The result of infected individuals is interesting and important. Please describe the details of RNA-seq.
A. Are they generated using total PBMCs or CD4+ T-cell population? If they are from PBMCs, different proportion of PBMC subset would affect the result. If they are from CD4 T cells, there is still different proportion of CD4 T cell subset between heathy donor and HTLV-1-infected individuals. Effector/memory CD4 T cell fraction is known to be increase in HTLV-1-infected individuals. The author had better mention the possibility.
B. How about the expression level of tax and HBZ in RNA-seq data? That would be informative to analyze relationship between Tax/HBZ and ASEs.

A. We thank the reviewer for this comment. In Figure 5, we have analyzed RNA-seq data from Kataoka et al. Nat. Genet. *47*, 1304–1315). These data were obtained from 35 ATL samples (17 acute, 11 chronic, 4 lymphoma, 2 smoldering, 1 unknown). These are samples isolated from peripheral blood, lymph nodes or tumor infiltrated tissues; PBMCs from 3 HTLV-1 asymptomatic carriers and 3 control samples, which are purified CD4+ T cells from healthy donors. Although this heterogeneity might affect our comparative analysis, it should be stated that the interpatient variability in cell composition is even larger within the ATL patient population, both for non-CD4 cells (NK cells, B-cells, monocytes, CD8 T-cells) and for CD4 subsets (CD4 naïve, CD4 memory (resting vs. activated), CD4 Treg, CD4 Tfh). As shown in the Figure below, total CD4 cells and CD4 subsets vary strongly and no single subset dominates across all patient samples (n=44). We mention these limitations in the discussion section (page 14, lines 21-25), and in the response to editorial comment#3. Most importantly, in the revised manuscript, we analyzed an additional cohort of Afro-Caribbean origin (Rosewick et al., 2017; Artesi et al., 2017; Marçais et al., 2021. The results are presented in the new Figure 6 and confirm the observation of alternative splicing perturbations in the first cohort. Therefore, the observation of validated splicing events (this study) and transcriptomic modules (Subramanian et al. 2019) across two genetically distinct populations, Japanese and Afro-Caribbean or Afro-Brazilian, respectively, argues in favor of conserved molecular mechanisms of ATL leukemogenesis.. Genes presenting ASEs in both cohorts are compiled in Table S3, sheet 18.

Figure Legend: Cellular heterogeneity in primary ATL samples (Kataoka et al. 2015) revealed by CIBERSORT cell type deconvolution, performed as in Subramanian et al. 2019.

C. As shown on the Figure below, in ATL samples the level of *Tax* in RNA-seq data compared to *HBZ,* is at least 10x less on average. However, there is a difference in expression kinetics of sense transcription (i.e *Tax* expression), which is heterogeneous and intermittent, and antisense transcription (i.e *HBZ* expression), which is more stable (Miura et al., PLoS Pathog. 2019 Nov 18;15(11):e1008164). We have added this information in the introduction section (page 5, lines 1-4), and discussion (page 14, lines 4-6). In addition, TPM data for *hbz* in ATL samples from the Afro-Caribbean cohort were reported in Rosewick et al. Suppl. Table 3 (Nat. Commun.*8*, 15264).

Figure legend: Number of RNA-seq reads from indicated samples (Kataoka et al. Nat. Genet. *47*, 1304–1315)

Reviewer #2: Major comments
Jurkat cells have an early T-cell phenotype and thus might not represent a good cell model to study HTLV-1 which infects and persists in mature T-cells. How do the levels of the proteins in the Jurkat system compare to the levels observed in productively infected cells or ATLL cells?

We thank the reviewer for this important question. Our detailed response is provided in the editorial comments responses section above (see #2).

The validation in patient samples is of key importance to assess the relevance of the results presented; however the cohorts examined are poorly matched in terms of numbers (35 ATLL, 3 HC, 3 normal controls), which is likely to skew the results. Furthermore there is no indication about the % of infected cells in the ATLL and HC samples. The comparison should be done between samples with similar % of infected cells, otherwise infected cells should be sorted.

We thank the reviewer for this question. Our detailed response is provided in the editorial comments responses section above (see #3). We have analyzed RNA-seq data from two independent, previously described cohorts (Kataoaka et al., Nat. Genet. *47*, 1304–1315. Rosewick Nat. Commun. *8*, 15264). We would like to stress that the effects of HTLV-1 infection as such are quantified in the analysis of healthy controls vs. asymptomatic carriers, whereas the effects of leukemogenesis (controls vs. ATL) strongly differ (Fig. 7). Moreover, sorting infected cells would not be possible since most ATL cells are Tax-negative and often contain partially deleted proviruses. Moreover, several ATL patients display >100% proviral load (caused by multiple integrations in the same leukemic cell clone), which precludes a simple “back-to-back” comparison of purified “infected” cells from ATL patients and asymptomatic carriers .

Reviewer #3: 1. The authors describe using ‘a mini-library of different Tax and HBZ clones with previously described functional domains’ on page 9. A brief description of the domains used in this study would be beneficial for the field. Also, was there sequence homology between the different clones for both Tax and HBZ?

We thank the reviewer for this comment. We now have shown, in a schematic representation, domains and point mutants used in this study (Figure S2). References for each are provided as Pubmed identifiers (PMIDs).

2. This study utilized an inducible cellular system to express Tax or HBZ, separately. What is the expression of inducible-Tax or -HBZ relative to naturally infected cells?

We thank the reviewer for raising this important question. Our response is provided in the editorial comments responses section above (see #2).

Are the effects observed through the interactome and transcriptome results long-lasting or transient (what would happen if you took away Tax or HBZ)?

We thank the reviewer for this interesting question. In another study, we are examining the possibility of long-lasting transcriptome effects of Tax. We have generated a Jurkat cell line harboring a *GFP* gene under the control of the HTLV-1 LTR5’, and containing a doxycycline inducible *Tax* gene. In this model, we induce the expression of Tax with doxycycline (I), monitor LTR5’ activation by examining GFP positive cells, sort GFP+ cells (II.). These GFP+ cells are then put back in culture for 10 days without Tax induction and progressively lose their GFP expression (III.). Finally, cells from step III. are re-induced for Tax expression for 2 days and GFP + cells are sorted.

We then performed single-cell sequencing of GFP+ and GFP- populations from both step I. and step IV. above, which clustered in 3 and 2 populations, respectively. Gene ontology (GO) analysis of identified genes indicated “Viral process, GO: 0016032” as one of the enriched terms from the first sorted GFP+ cells. Interestingly, following Tax silencing (step III) and re-induction, viral latency (GO:0019043) and proviral latency (GO:0075713), which are “child” terms of viral process, were enriched. We thus speculate that some changes induced by Tax in the gene expression pathway, such as expression of viral latency genes, are indeed long-lasting. Other events are transient as suggested by our comparison of both GFP+ populations (see figure below). Inspired by this reviewer’s question we plan to perform validation experiments that could quantify transient versus permanent Tax or HBZ effects on gene expression.

3. In these experiments, was the hbz mRNA also present? Hbz mRNA has been shown to have proliferative effects. It’s difficult to say whether the transcriptome results found were solely due to the protein, the mRNA or both.

We agree with this comment. In our experimental setting, HBZ is expressed from a cDNA sequence cloned into a lentiviral vector. As shown above in Figure S4A-D, HBZ mRNA is detected in Jurkat cells at levels which overlap with ex vivo HBZ mRNA levels in ATL patients. Since it is logistically too challenging to quantify HBZ protein in large numbers of primary ATL samples, we indeed cannot distinguish between biological effects due solely to HBZ protein, mRNA or both. However, our combined in vitro and ex vivo analyses show that Jurkat HBZ mRNA is expressed at physiologically relevant levels that mimic the in vivo situation in ATL, whereas Tax mRNA in our Jurkat model might mimic the in vivo situation in HAM/TSP (Fig. S4A).

Part III: Minor issues:

Reviewer #1: 0. There are several previous papers about HTLV-1 and RNA splicing (PMID 25519886, PMID 3254671, PMID 23600753). The authors should use them as references and discuss current study results by considering previous reports.

We thank the reviewer for this comment. There are several interesting overlaps between our study and these previous reports.

1. In both our study and Ben Ameur et al. (Nat. Comm. 2020 Jun 16;11(1):3045), (i) the majority of Tax-dependent alternative splicing events are skipped exons (compare Fig 1b from Ben Ameur et al. and this study Fig.3B “Tax”); (ii) a small number of genes are spliced co-transcriptionally following Tax expression (compare Fig 1a from Ben Ameur et al. and this study Fig. 4D, see also our response to reviewer#1, question #2 above). Importantly, these studies use different cell lines to analyze Tax effects (HEK293 in Ben Ameur et al.; Jurkat in this study), and indicate a general molecular perturbation of the host cell gene expression pathway.

2. We now highlight in our discussion (page 16, lines 1-5), the fact that a number of genes presenting alternative splicing in untransformed infected CD4+ clones and ATLL samples (Thenoz et al. [Retrovirology.](https://www.ncbi.nlm.nih.gov/pmc/articles/PMC4293115/) 2014; 11: 119), are also shared in our dataset from Tax and HBZ expressing Jurkat cells.

3. Splicing of members of the IKaros family of zinc-finger protein coding genes have been reported previously by Asanuma et al., ([Cancer Sci.](https://www.ncbi.nlm.nih.gov/pmc/articles/PMC7657222/) 2013 Aug; 104(8): 1097–1106). While their study showed abnormal splicing mostly in the Helios gene (*IKZF2*), our study identified different alternative splicing events in *IKZF1, IKZF2, IKZF3 and IKZF5* in Jurkat cells expressing Tax or HBZ*;* as well in the two patient cohorts samples examined (see Table S3). Additionally, we detected differential expressions of *IKZF3* (log2FoldChange: 1.037) and *IKZF4* (log2FoldChange: 1.847) following Tax expression in Jurkat cells (Table S2).

These comparisons, between our data and previously published data, have the potential to identify potential ATL biomarkers such as the IKaros family of zinc-finger protein coding genes or the *ITGAE* (coding for integrin alpha E or CD103). We have discussed this hypothesis (page 16, lines 1-5) .

1. Fig. 1A-C can be shown as supplementary figures.

We agree with this comment. Previous Fig. 1 A-C is now presented as supplemental figures (Fig. S1A-C).

2. Fig. 1E: What Overlap and Union means?

Previous Fig. 1E is now Fig. 1D. Overlap means interactions already known from the literature and retrieved from our Y2H screen (Table S1, compare Y2H_2020 and other datasets). We found 9 overlapping partners for Tax and 5 for HBZ. Union means the current updated list of interactions combining our study and information from the literature. We have clarified these terms in the figure legend (Page 26, lines 20-21).

3. Fig. 1G: There is no description in the paper. If so, please delete that.

Previous Fig. 1G, which is now Fig. 1F shows the distribution of the 34 cancer gene products into either Tax, HBZ or both protein interactors.

4. Fig. 2E-F: Please check again if “P=0.00” or “q = 0.00” is correct or not.

This annotation means that the p or q value is less than 0.001 (corresponding to 1/the number of permutations=1000). The p and q values on Fig. 2E-F have been modified accordingly.

5. Fig. 3E: Please provide more information how they analyzed and showed the figure.

Fig. 3E from the previous version is now Fig. 4A, and it is presented as bar graphs for more clarity as suggested by this reviewer. We show, side-by-side, ΔPSI calculated from RNA-seq data using rMATS and ΔPSI calculated from RT-PCR validation experiments. 22 out of 23 tested spliced events exhibited a similar tendency of splicing (inclusion or exclusion). Moreover, we have added information on how the ΔPSI(RT-PCR) is calculated in the methods section (Page 23, lines 31-33 and Page 24, lines 1-4).

6. Fig. 6: It is not clear how to see this figure. There seems to be little graphical difference. Is the difference is statistically significant?

These events have been evaluated as statistically significant by rMATS (FDR <0.05). The sashimi plots allow to visualize the splicing events but it is indeed not convenient to observe PSI differences between conditions. Previous Fig. 6 is now presented as a supplemental figure (Fig. S7), and we show in main figures (current Fig. 7 B-F), box plots that clearly show PSI differences between controls and cells expressing Tax or HBZ; or controls and HTLV+ samples.

Previous Figure 6 (Now Fig. S7)

**Revised Figure 7 B-F**

7. Fig. 7C: Please explain more in details. Which molecules are associated with Tax or HBZ?

Previous Fig. 7C is now Fig. 8C. The figure indicates RNA-binding proteins (RBPs) for which binding motifs are enriched in ASEs detected in ATLL samples. Sequences of regulated exons and part of their flanking introns were screened for RNA-binding motifs using AME from the MEME suite. Enriched motif(s) of RBPs with an adjusted p-value <0.05 are shown. Identified partners of Tax (U2AF2) or HBZ (A1CF) are underlined in red. We have modified the figure legend (page 29, lines 1-3).

8. Fig. 7E: The result is not convincing. I suggest to revised the figure.

Previous Fig. 7E is now Fig. 8E. The immunoprecipitated Tax band, which is absent in RNase treated samples, is now indicated by an arrow.

Previous Fig. 7E

Revised Fig. 8E

9. Table S2: 12 genes whose expression was altered in opposite directions between Tax and HBZ are interesting. It would be useful to show the list.

The list has been added to the supplementary Tables S2, sheet 7.

Reviewer #2: Additional comments
There are no page and line numbers

Page and line numbers are now shown.

The title of the manuscript should specify ‘HTLV-1’

The title is now changed to “ **The HTLV-1 viral oncoproteins Tax and HBZ reprogram the cellular mRNA splicing landscape”**

The first sentence of the abstract starts with ‘while’. This is poor English.

The first sentence is changed to “Viral infections are known to hijack the transcription and translation of the host cell”

In the Synopsis, the first statement should specify ‘HTLV-1-encoded proteins Tax and HBZ’ and not the second.

The synopsis was replaced by an “author summary” to comply with Plos pathogens editing.

First paragraph of the Introduction: Hayward et al., 1981 is not in the reference list.
Second paragraph of the Introduction: The review by Matsuoka and Mesnard on HBZ (Retrovirology 2020) and by Mohanty and Harhaj on Tax (Pathogens 2020) would provide more up to date information compared to the cited reviews.

We agree with the reviewer and we have updated the references (Page 4 lines 20-21).

Results - First section:
Figures 1A, 1B and 1C are very simplistic and could be removed or placed in the supplemental section.

These Figures have been moved to supplemental Figure S1.

Some of the Tax mutants used in the yeast experiments predate the references provided in the text. For example, M10, M22 and M47 were described by Smith MR and Greene WC (Genes Dev 1990). Table S1 should provide a brief description of these and other mutants whose names are not self-explanatory. It would also be helpful to include a diagram of the domain structures of Tax and HBZ so that the reader understands the locations of the Tax mutations and HBZ domains tested. Figure 1E presents information that is not important. It could be removed or moved to the Supplemental section.

A diagram of Tax and HBZ domains and mutants used in this study has been added as supplemental Figure 2. The references for Tax and HBZ mutants used are now provided as PMIDs.

Figure S1 lacks a description of panels D and E. How many repeats were performed? What do the error bars represent?

We would like to apologize for these omissions. Previous Figures S1 D and E are now presented as Fig. 1 B-C, and the description is provided in the legend (Page 26, lines 16-19).

The paragraph describing Figure 1F highlights that Tax and HBZ have many more PPIs compared to known cancer gene products or other tumor virus proteins, presumably based on 2 cited references from 2014 and 2012, respectively. It is likely that more interactions have been described in the intervening years, so the relevance of this comparison is questionable.

We thank the reviewer for this comment. Based on the human reference interactome dataset (HuRI, [www.interactome-atlas.org](http://www.interactome-atlas.org)) that we published last year (Luck et al. 2020, *Nature* **580,** 402–408), the average “degree” (number of binary interactors) of cancer gene products is 13.4. However, as shown below some cancer gene products such as CREB3L1, CD79A, REL, TRIM27, LMO2 and MUC1 have also at least 100 reported PPIs

Regarding tumor viruses, to our best of knowledge, there is no recent unbiased systematic study analyzing tumor virus PPIs since the publication of Rozenblatt-Rosen et al. (Nature. 2012 Jul 26;487(7408):491-5.). The average “degree” for human tumor viral proteins analyzed is 12.9. However, some proteins such as E6, E7 from different HPV strains exhibit a degree of 53 and 117, respectively. We have revised the sentence accordingly (Page 6, line 34; page 7 lines 1-2).

Results Second section:
The statement that Tax and HBZ are differentially expressed in vivo is not accurate, in particular if one considers infected cells, and not ATLL cells. There is no need to use this argument to justify the use of Jurkat cells expressing the individual proteins.

We agree with this reviewers’ comment and have removed the sentence.

In Figure S3A, the levels of the housekeeper protein HSP70 are very different. When this difference is compared to the levels of Tax and HBZ detected, the difference in the levels of these proteins becomes enormous. How do the levels of the proteins in the Jurkat system compare to the levels observed in productively infected cells or ATLL cells?

In response to reviewer and editorial comment #2, we have proposed new Figures S4A-D that allows a more accurate estimate of Tax or HBZ expression in our inducible system compared to HTLV-1 positive primary cells.

According to the Methods section, the confocal analysis was carried out in HeLa cells but the legend to Figure S3D refers to HEK293T cells.

We would like to apologize for these errors. They have been corrected.

This needs to be corrected. The images shown in Panel E are not convincing. In general, it would have been preferable to carry out these experiments in a cell system that better approximates mature T-cells. Also, the title of this figure does not describe Panels A-C. In Panels B and C ‘Normalized’, not ‘Noralized’. What do the asterisks mean?

We would like to apologize for these errors. They have been corrected. The asterisks mean p-values (now indicated in the figure legend).

Results - Third section:
The first sentence talks about transcriptional ‘defects’. This should read ‘effects’. The information shown in Figure 3A is not essential. Figure 3E is cryptic. It would be more helpful to show the results of the RT-PCR assays of the individual genes as bar graphs.

We thank the review for these comments. Figure 3A helps readers not familiar with analyzing alternative splicing events. Fig. 3E from the previous version is now Fig. 4A (also shown above in response to reviewer #1 major question#1), and it is now presented as bar graphs for more clarity as suggested by this reviewer. We show, side-by-side, ΔPSI calculated from RNA-seq data using rMATS and ΔPSI calculated from RT-PCR validation experiments. 22 out of 23 tested spliced events exhibited a similar tendency of splicing (inclusion or exclusion).

Results - Fourth Section:
Figure 4B should list the genes in alphabetical order.

The 2 panels in Figure 4C should labeled ‘Tax’ and ‘HBZ’ to help the reader.

Previous Figure 4C is now Fig. 4D and is labeled as suggested by this reviewer.

The title to Figure S4 should be the same as the one that appears in the figure.

This has been corrected.

Are the ASes listed in the figure shared among the 3 types of samples? As written, this is unclear.

We agree with this comment: previous Fig. S4, which is now Fig. S5, shows a list of shared ASEs.

The title to Figure S5 does not describe panels B and C.

Previous Fig. S5 is now Fig. S7 and the title was corrected.

What do the lines in the bars represent? Means or Medians? They look identical for unchanged vs excluded. The p values don’t seem to correspond to the differences as visualized in the figure.

The lines represent the medians, we have added this information in the legend. Yes indeed the means of the two conditions are 12.14145 and 12.4405, respectively. This difference is still significant as the calculated p-value was below 0.05 using Welch Two Sample t-test.

Results - Fifth Seciton:

The pie chart in Figure 5A might have a problem - the section representing MXE (19.5%) seems to cover about 1/3 of the pie.

This has been corrected.

Results - Sixth section:
Figure 7C indicates HuR. The proper gene symbol is ELAVL1. Official gene symbols should be used in the figures; common names can be included in the text.

This has been corrected.

Reviewer #3: 1. The ‘Synposis’ section was somewhat confusing without a figure legend describing the image.

The “synopsis” figure was removed to comply with Plos Pathogens editing standards

2. Figure 1B: HBZ has been shown to interact with CREB and prevent it from binding to the viral LTR CRE binding sites (i.e. sequesters CREB away from promoter). The diagram shown here suggests the complex is bound at the chromatin and inhibits transcription.

We thank the reviewer for this comment. Previous Figure 1B is now Fig S1B. The diagram was corrected.

3. In Figure 7E, the authors should distinguish the background band (upper band?) and Tax band in the Tax western blot. Also, in what cells were these experiments performed?

Previous Fig 7E is now Fig 8E. An arrow has been added to indicate the Tax band. We also have added the cell line used in the legend (Page 29, line 5).

4. Could Tax or HBZ have any effect on viral RNA splicing?

Our study focused on the interaction of Tax and HBZ with splicing regulators and the influence on host gene splicing. Nevertheless, most viral pre-mRNAs need to be spliced and the HTLV-1 genome possesses numerous alternative splice sites. Moreover, Thenoz *et al.* showed that inhibition of alternative splicing by Sudemycin E could reduce Tax protein levels as well as HBZ gene expression (Thenoz et al. [Retrovirology.](https://www.ncbi.nlm.nih.gov/pmc/articles/PMC4293115/) 2014; 11: 119). It is therefore likely that Tax and HBZ exploit the splicing machinery to regulate alternative splicing events of viral pre-mRNAs, including their respective exons.

HTLV-1 splice sites are well defined, however the regulation of alternative splicing during viral gene expression has not been extensively studied, except the well-known role of Rex, which is able to directly or indirectly repress viral intron splicing (Gröne M et al., Virology. 1996 Apr 15;218(2):316-25) . HTLV-1 RNAs have been mostly studied in the context of their expression levels (Seiki et al. Virology. 1990 May;176(1):81-6.; Cavallari et al., J Virol. 2015 Nov 18;90(3):1486-98).

We have discussed potential implications of the U2AF complex in the regulation of HTLV-1 viral RNA splicing (page 16, lines 21-22).
