# Supplementary material for: The HTLV-1 viral oncoproteins Tax and HBZ reprogram the cellular mRNA splicing landscape
Source: PLoS Pathog. 2021 Sep 20;17(9):e1009919. doi: 10.1371/journal.ppat.1009919 (PMC8483338; doi:10.1371/journal.ppat.1009919)
Supplement: Attachment — Submitted filename: Responses to reviewers #2.docx [file ppat.1009919.s013.docx]

# **Responses to reviewers (in blue below each comment)**

While two reviewers are content with your response to the points raised, I agree with Reviewer 2 that severe limitations remain in the reliance on Jurkat cells to infer normal physiological actions of the viral proteins. I would therefore ask you to clearly and explicitly acknowledge the limitations of this approach in the Discussion section; the current text at lines 9-12 on p.14 refer to the interplay between the viral proteins, rather than to the nature of the cell line or the potentially unphysiological level of expression of the proteins. Second, lines 4-6 on p.14 are potentially misleading: it is not clear whether the phrase 'in the initial steps of HTLV-1 infection' concerns only ATLL cells or non-malignant infected cells as well, and whether it refers to primary infection of the host or to new infection of a single cell. Please clarify this sentence.

We thank you for the above comments. First, we agree with you and reviewer 2 that Jurkat cells, like any other *in vitro* culture system, has significant limitations especially when it comes to draw conclusions in the context of primary cells. Second, because we are using vectors to produce Tax and HBZ in Jurkat cells, while in primary infected T-cells these viral products are expressed in the context of an integrated HTLV-1 provirus and other viral proteins/transcripts, we acknowledge that differences in the expression levels between experimental systems may require caution for the interpretation of the data. We acknowledge that this was not sufficiently highlighted in the latest version of our manuscript and have modified the Discussion to improve this point as suggested above.

More specifically, we have clearly and explicitly acknowledged the limitations of using Jurkat cell to infer normal physiological actions of the viral proteins (p14, lines 4-6 and 9-15).

However, we do also note that there are over 300 manuscripts found in PubMed that utilize Jurkat cells to study Tax and/or HBZ functions. Lines 9-12 and 4-6 on page 14 are revised as follows:

Lines 9-15 in the revised manuscript:

“Jurkat is an established T-cell acute lymphoblastic leukemia cell line ([Schneider](https://pubmed.ncbi.nlm.nih.gov/?sort=date&term=Schneider+U&cauthor_id=68013) et al. 1976), which, like any *in vitro* culture system, carries genomic and phenotypic differences when compared to primary T cells isolated from healthy donors or HTLV-1 infected peripheral blood mononuclear cells. Although our Jurkat experimental system may be limited in capturing the *in vivo* physiological effects of Tax and HBZ in primary cells, it does allow for a systematic comparative analysis for identifying Tax- and HBZ-dependent events contributing to the dysregulation of cellular functions.”

Lines 4-6 on page 14 are revised as follows:

“In HTLV-1 positive cells, the *HBZ* antisense transcript is consistently expressed while Tax-dependent sense transcription occurs in a burst-like manner, allowing HTLV-1 transmission to naïve T- cells (Miura et al., 2019).”

Reviewer #2: Main points:

1 - The Authors present a legthy justification for using jurkat cells as a model of HTLV-1 infection/persistence, however this cell line remains suboptimal for HTLV-1 studies.

We thank the reviewer for this comment, and we have acknowledged the limitations of using Jurkat cells as a model of HTLV-1 infection (please see the above response to editors’ comments).

2 - The expression levels of Tax and HBZ measured by the Authors in Jurkat cells differ greatly from those estimated in ATL cells (137-fold higher for Tax and 50-fold lower for HBZ), suggesting that the effects observed in the jurkat model might not faithfully represent what happens in patients. The Authors' justification that the intra-group variability in patients is also high is not a strong argument.

We agree with this comment and tried to address this issue by highlighting the limitations of our *in vitro* system in the revised version (also see our comment in response to the editor’s concerns above). The goal of the *in vitro* section of our study (Jurkat cells) is not to replicate the expression levels of Tax and HBZ observed in a physiological context. The model rather allows us to investigate Tax- and HBZ-dependent events contributing to the dysregulation of transcription and post-transcription events.

3 - The validation studies in patients are still weakened by the poor match between controls and patients. Instead of testing a larger number of controls, the Authors analyzed in silico data from patients of different ethnicity/geographical origin. This is a rather convoluted and indirect approach. It would have been much better to actually test an appropriate number of controls.

Our validation studies were performed using previously published datasets (Rosewick et al 2017 and Marçais et al 2021; Kataoka et al. 2015). Although we agree that additional healthy individuals would have been an asset, we like to point out that the Afro-Carrabean dataset added in the revised manuscript consists of both infected individuals and healthy controls of different ethnicity. We have acknowledged the limitations of these two datasets in terms of healthy controls in our discussion section, Lines 26-28, page 14.

Lines 26-28, page 14 in the revised manuscript:

“These two cohorts are unbalanced in the number of control samples from healthy individuals (purified CD4+ T-cells) compared to HTLV-1 positive samples, and their genetic and epidemiological heterogeneity may affect our results”
